# Trauma, Resilience, and Mental Health in Migrant and Non-Migrant Youth: An International Cross-Sectional Study Across Six Countries

**DOI:** 10.3389/fpsyt.2019.00997

**Published:** 2020-03-09

**Authors:** Justine M. Gatt, Rebecca Alexander, Alan Emond, Kim Foster, Kristin Hadfield, Amanda Mason-Jones, Steve Reid, Linda Theron, Michael Ungar, Trecia A. Wouldes, Qiaobing Wu

**Affiliations:** ^1^ Neuroscience Research Australia, Randwick, NSW, Australia; ^2^ School of Psychology, University of New South Wales, Sydney, NSW, Australia; ^3^ Centre for Academic Child Health, University of Bristol Medical School, Bristol, United Kingdom; ^4^ School of Nursing, Midwifery and Paramedicine, Australian Catholic University, Melbourne, VIC, Australia; ^5^ Department of Biological and Experimental Psychology, Queen Mary University of London, London, United Kingdom; ^6^ Department of Health Sciences, University of York, York, United Kingdom; ^7^ Primary Health Care Directorate, University of Cape Town, Cape Town, South Africa; ^8^ Department of Educational Psychology, University of Pretoria, Pretoria, South Africa; ^9^ Resilience Research Centre, Dalhousie University, Halifax, NS, Canada; ^10^ Department of Psychological Medicine, The University of Auckland, Auckland, New Zealand; ^11^ Department of Applied Social Sciences, The Hong Kong Polytechnic University, Hong Kong, Hong Kong

**Keywords:** trauma, resilience, mental health, migrant, youth, wellbeing, COMPAS-W, CYRM-28

## Abstract

Resilience is a dynamic process of positive adaptation to significant adversity. While there has been substantial focus on risks and negative outcomes associated with youth migrancy, there is limited evidence of the relationship between the adversity of migration, and resilience, wellbeing, and positive mental health in adolescents. This international study aimed to explore the differences in resilience, wellbeing, and mental health behaviors in migrant and non-migrant adolescents tested across six countries (Australia, New Zealand, UK, China, South Africa, and Canada) with varying levels of trauma exposure. The study was a cross-sectional survey design with a convenience sample of 194 10–17 year old migrants and non-migrants. The migrant sample included both “internal” migrants (change of residence within a country) and “external” migrants (change of residence across national borders) for comparison. Across the sites, migrants reported a higher mean number of traumatic events for the past year than non-migrants, with internal migrants reporting more events than external migrants overall. South African adolescents reported a higher mean number of traumatic events for the past year than all other sites. External migrants reported higher resilience scores yet reduced prosocial behaviors relative to internal migrants and non-migrants, whereas both internal and external migrants reported higher peer problems than non-migrants. When considering the interacting effects of trauma, the presence or absence of trauma did not appear to impact migrant scores in terms of resilience, wellbeing, or conduct problems. In comparison, trauma-exposed non-migrants showed detriments relative to trauma-exposed migrant peers for all of these measures. In conclusion, the survey tool was found to be reliable and acceptable for use in international studies of different samples of adolescent migrants. Overall, migrant adolescents showed greater resilience resources than non-migrants and, although the migrants experienced more traumatic events, the impact of trauma on mental health outcomes was greater in the non-migrants. There is a need for further research with larger prospective sample sizes to investigate how levels of resilience and wellbeing vary over time and across countries, and the ways resilience can be promoted in adolescents exposed to trauma, regardless of migrancy status.

## Introduction

Understanding the mechanisms that underpin resilience to trauma is a surging field of enquiry in mental health research, particularly in adolescents. The impact of migration is another public health challenge and is sometimes precipitated by adversity experienced in the home country or region. Worldwide there are approximately 35 million migrants between the ages of 10 and 24, which represent 17% of the total migrant population. Of those, 9 million (25%) are in the 10–14 year age group and 11 million (32%) are in middle to late adolescence (15–19) (1). There are two basic types of migration; internal and external. Internal migration usually refers to a change of residence within a country such as movement from rural to urban settings or movement from state to state. External migration refers to a change of residence over national boundaries or moving to a different country. External migrants can be further classified into people who followed legal and illegal migrant routes, and refugees. The motivation for these different types of migration often differs, and which can provide diverse challenges to the migrant before and after their arrival in their new home (2, 3). However, current research is unclear as to whether there are common challenges for internal and external adolescent migrants and how these challenges may affect adolescence and the transition from childhood to adulthood during this crucial stage of development (4–6). This is unfortunate because adolescence is a key decade in the life-course where physical health, mental health, and behavioral problems can arise that will have an ongoing impact throughout adulthood.

Many of the risk factors for mental health and behavioral problems begin during adolescence and include tobacco use, harmful use of alcohol and cannabis, and unhealthy diets (7). The onset of mental disorders such as depression and anxiety disorders typically occur in childhood and adolescence, with 20% of the world's children and adolescents experiencing mental disorders, half of those beginning prior to age 14 (8). Left untreated, these conditions can severely impact development, educational attainment, and place young people at higher risk of suicide (9). Substance abuse, conduct problems, and mental disorders in adolescence are often triggered by psychological trauma, either by direct experience of a traumatic event such as interpersonal violence or through secondary traumatic stress that occurs when a close family member or friend has experienced a traumatic event (10, 11). The kind, number, and complexity of traumas experienced in early life have a differential impact on psychological and behavioral difficulties (12, 13). In addition, children exposed to trauma may continue to develop new symptoms over time as they encounter additional developmental or environmental challenges and stressors (14–16). Yet, it is still unclear as to why some children exposed to trauma develop emotional and behavioral problems while others do not (11).

Resilience as a construct is the process of positive adaptation and/or recovery from trauma or adversity (17). Multiple systems are understood to interact to provide the resources required for resilience (18, 19). Factors that have been associated with resilience in childhood and adolescence, include positive caregiver, family and peer relationships, religion, school environment, and personal characteristics such as self-regulation and coping skills (11, 20, 21). Low resilience to adversity puts individuals at higher risk of developing psychiatric problems with depression, anxiety, and conduct disorder being the most common (22, 23).

Research in adolescent migrants have identified protective factors for mental health, suggestive of resilience processes (2, 3, 24–26). In one study, pre-migration poverty combined with clandestine entry in the United States increased the risk for symptoms related to post-traumatic stress disorder (PTSD) (2). Post-migration discrimination and poor neighborhoods also increased the risk for PTSD whereas a positive family environment and social support mitigated risk (2). In a review of the mental health of refugee children resettled in high-income countries, risk of developing mental health problems was associated with trauma exposure, parental exposure to violence, loss of parent(s), limited family support, violence and discrimination in the host country, feeling disconnected to school, and neighborhood violence (25). Protective factors included stable settlement and social support in the host country, psychological wellbeing of the parents/guardians, and religious beliefs (25). Overall however, most studies have largely focused on vulnerability or risk in refugee populations relative to non-migrants with little focus on comparisons with immigrant youth, or within immigrant groups defined more broadly (e.g., immigrant youth who migrated at some undefined point in time, and/or second-generation immigrant youth with first-generation immigrant parents), with most, if not all, studies conducted within the one country, with no comparison across multiple country sites (27–30).

Recognizing the gaps in our understanding of mental health in adolescent migrants, an international collaboration was established through the Worldwide Universities Network to investigate resilience (31). The aim of this collaboration is to establish a longitudinal study that would identify the mechanisms or processes that promote physical and mental wellbeing and prevent mental illness despite exposure to the adversity brought about by adapting to a new culture and the challenges of transitioning through adolescence. This collaboration includes a multidisciplinary group of researchers from Australia, Canada, China, New Zealand (NZ), South Africa, and the United Kingdom (UK). Through this collaboration a questionnaire battery was designed and piloted in these countries with the intention of comparing the resilience of adolescent migrants with non-migrants. The questionnaires were based on an in-house literature review of resilience in adolescent migrants, and qualitative data collected during focus groups in the NZ, South Africa, and the UK. The sites chosen for focus group discussions offered diverse contexts for the study, and were linked to the Worldwide Universities Network and had the resources and expertise to conduct qualitative interviews.

This aim of this report is to use our pilot data to explore the impact of country-specific factors, migrancy, and trauma exposure on resilience, wellbeing, and mental health among migrant and non-migrant adolescents aged 10–17 in countries where there are high rates of internal and external migration. The overall hypotheses are that migrants and non-migrants might vary in behavior and mental health outcomes by virtue of differences in exposure to trauma and adversity, and that higher resilience would be associated with better wellbeing, fewer symptoms of mental illness, and fewer behavioral problems. The specific questions addressed in this study are the following: (1) are the measures of resilience, wellbeing, mental health, and behavior reliable across country sites? (2) do differences exist between migrant and non-migrant adolescents (controlling for any site differences) in trauma exposure? (3) are there differences between migrants and non-migrants in behavioral and mental health outcomes? and (4) how is trauma and migration related to resilience, behavior, and wellbeing?

## Materials and Methods

This pilot study, conducted across six countries: Australia, Canada, China, NZ, South Africa, and the UK, used a cross-sectional survey design with a convenience sample of 194 10–17 year-old migrant and non-migrant youth. Migrants included internal migrants who had moved within a country, and external migrants who had moved across national borders.

### Participants

The sample comprised 194 adolescents from: Australia (*n* = 25), Canada (*n* = 21), China (*n* = 77), NZ (*n* = 33), South Africa (*n* = 28), and the UK (*n* = 10). Participants ranged in age from 10 to 17 years (*M* = 13.9, *SD* = **1.36), with the sample made up of 52% males (*n* = 101), 46% females (*n* = 89), and 2% sex undisclosed (*n* = 4). Within the sample, 77% of participants were migrants and 23% were non-migrants. **Table 1** contains a breakdown of migrant status across research sites.

**Table 1:** Age, sex, and migrant status by site.

Site	N	Age (mean ± SD)	Age range	Sex (N, %)	Migrant status	Country of birth (majority)
Australia	25	13.3 (0.61)	12–14 years	M: 17 (68%) F: 8 (32%)	Migrant: 0 Non-migrant: 25	n = 0 Australia (n = 24)*
Canada	21	14.1 (0.97)	13–15 years	M: 8 (38%) F: 13 (62%)	Migrant^E^: 21 Non-migrant: 0	Iraq (n = 9)** n = 0
China	77	13.2 (0.96)	12–17 years	M: 44 (57%) F: 29 (38%)	Migrant^I^: 77 Non-migrant: 0	Guangzhou, China (n = 25)*** n = 0
New Zealand (NZ)	33	15.3 (1.11)	12–16 years	M: 9 (27%) F: 24 (73%)	Migrant^E^: 19 Non-Migrant: 14	Philippines (n = 10)**** New Zealand (n = 19)
South Africa (SA)	28	13.8 (1.58)	10–16 years	M: 19 (68%) F: 9 (32%)	Migrant^I^: 28 Non-migrant: 0	South Africa (n = 20)***** n = 0
United Kingdom (UK)	10	15.7 (1.25)	13–17 years	M: 4 (40%) F: 6 (60%)	Migrant^E^: 4 Non-migrant: 6	Europe (n = 3)***** England (UK) (n = 6)
**TOTAL**	**194**	**13.9 (1.36)**	**10–17 years**	**M: 101 (52%)** **F: 89 (46%)**	**Migrant: 105^I^, 89^E^** **Non-migrant: 45**	**Guangzhou (n = 25), SA (n = 20)** **Australia (n = 24), NZ (n = 14)**

M, male; F, female; migrant^E^, external migrant (cross-country); migrant^I^, internal migrant (within-country). Country of birth origin: *Australia non-migrants: 24 Australia, 1 USA; ** Canada external migrants: 9 Iraq, 2 Australia/China/Uganda, 1 Syria/Yeman/Nepal/Congo/Qatar/Pakistan; *** China internal migrants: 25 Guangzhou, China, 43 “other”; **** New Zealand external migrants: 10 Philippines, 4 England (UK), 2 China, 1 Oman/Malaysia/India; ***** South Africa internal migrants: 20 South Africa, 3 Congo, 2 Zimbabwe, 1 Burundi/Mozambique; ***** UK external migrants: 1 the Netherlands, 1 France, 1 Poland, 1 USA.

Youth were recruited from schools (Australia, UK, China), youth centers (South Africa), an after-school program for migrants (Canada), or community networks (New Zealand) (**Table 1**). Details regarding participant recruitment per site are as follows. In Australia, head teachers from several independent NSW schools were approached for study participation. For participating schools, the head teacher forwarded study information to students and their parents for written consent. Head teachers then organized testing days and times for students to complete the questionnaires during school hours with a research team member. In the UK, youth were recruited from two state secondary schools in Bristol: after written informed consent was obtained from a parent, the students completed the questionnaires during school hours with a research team member. In China, youth were recruited from one secondary school in the city of Guangzhou, Guangdong province, where many migrants concentrate. The school principal helped select one class randomly from each of the three grades (grade 7^th^–9^th^), collected informed consent from the students and their parents, and arranged the time for students to complete the survey in class, with the presence of a research team member. In South Africa, youth center staff acted as gatekeepers. They advertised the study and provided any interested youth with consent forms (which needed to be co-signed by a parent/caregiver). In Canada, participants were sampled through an after-school program run by the YMCA Centre for Immigrant Programs. An information sheet and consent form was sent to all parents of children in the program and then those children with a completed consent form were able to participate in the study. Students completed the questionnaire during the after-school program time. And in New Zealand, families with adolescents in the target age group were identified through advertisements posted in community centers and through Worldwide Universities Network (WUN) research staff and student networks.

Ethics approval was sought and gained from the respective sites according to the local Human Research Ethics Committee processes (Australia; University of New South Wales Human Research Ethics Committee: HC15672; Canada; Dalhousie University Social Sciences and Humanities Research Ethics Board: REB 2015-3666; China; Chinese University of Hong Kong; New Zealand; The University of Auckland Human Ethics Committee: 015968; South Africa: North-West University Humanities and Health Research Ethics Committee: NWU-HS-2015-0234; United Kingdom; University of Bristol Faculty of Medicine Research Ethics Committee: ref 2016/26061). Written and/or verbal information was provided to all participants. Informed verbal and/or written consent was obtained from parents and informed verbal or written assent was gained from youth.

### Measures

**Figure 1:**
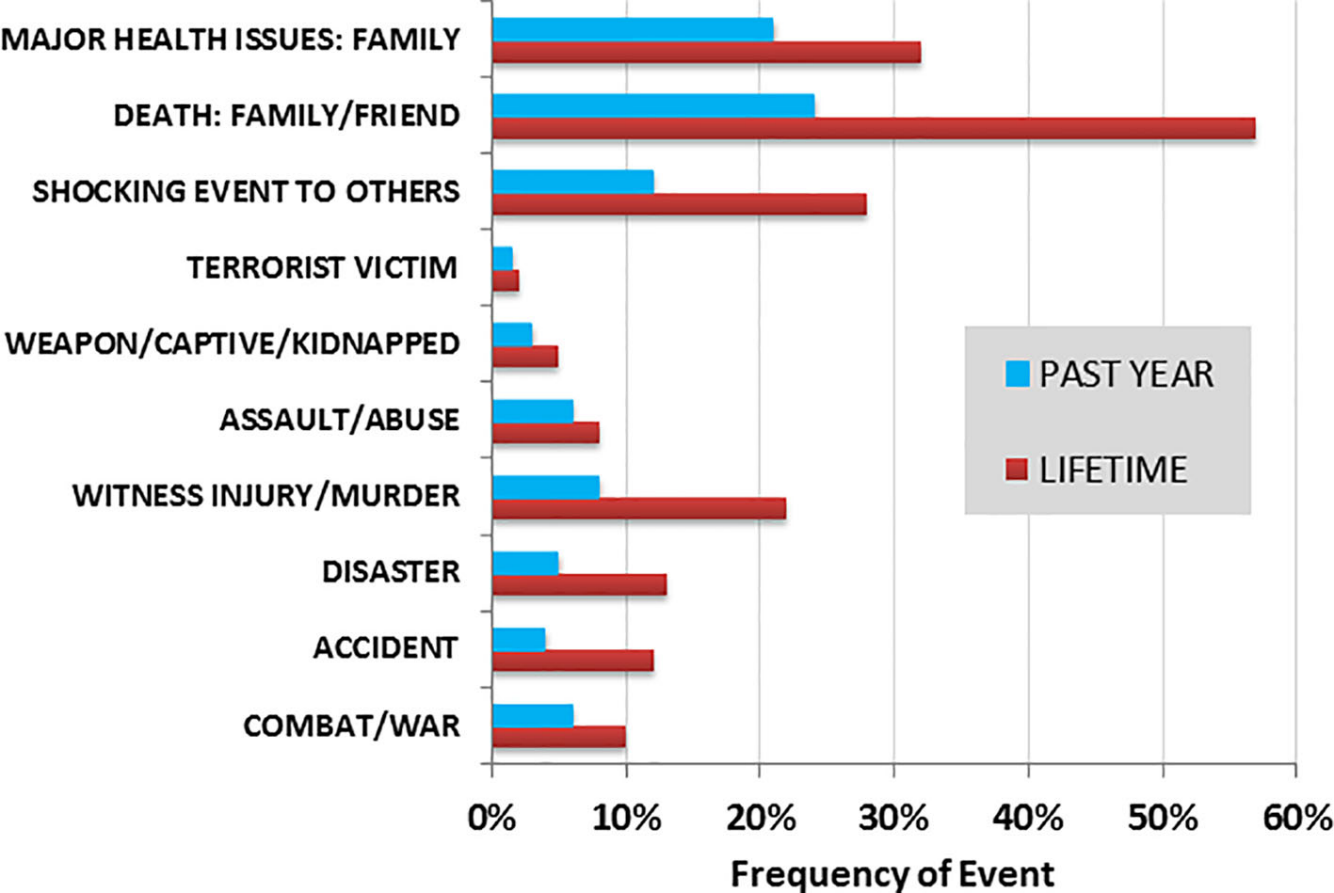
Frequency (%) of childhood trauma exposure reported across the sample for the past year and lifetime (*N* = 194). The corresponding question items for each of the trauma categories are as follows: i) combat/war (“have you ever had direct combat experience in a war?”); ii) accident (“have you ever been involved in a life-threatening accident?”); iii) disaster (“have you ever been involved in a fire, flood, or other natural disaster?”); iv) witness injury/murder (“have you ever witnessed someone being badly injured or killed?”); v) assault/abuse (“have you ever been seriously attacked or assaulted?”); vi) weapon/captive/kidnapped (“have you ever been threatened with a weapon, held captive, or kidnapped?”); vii) terrorist victim (“have you ever been the victim of terrorists?”); viii) shocking event to others (“have you suffered a great shock because one of the events on the list happened to someone close to you?”); ix) death: family/friend (“have you experienced the death of a close family member or close friend?”); and x) major health issues: family (“have you experienced a major change in health or behavior of a family member?”).

A questionnaire battery was developed using established measures from the literature and information derived from qualitative focus groups with youth in three of the participating countries. The questionnaire commenced with a series of demographic questions (e.g., gender, country of birth, ethnicity), followed by questions about participants' family structure, schooling experiences, neighborhood, personal and familial health, as well as trauma exposure using items adapted from the Early Life Stress Questionnaire (32) (see **Figure 1** legend for a list of trauma exposure items). The battery also contained the following measures: 1) Child and Youth Resilience Measure (CYRM-28) (33); 2) Connor-Davidson Resilience Scale (CD-RISC) (34); 3) Warwick-Edinburgh Mental Well-being Scale (WEMWBS) (35); 4) COMPAS Wellbeing Scale (COMPAS-W) (36); 5) Depression, Anxiety, Stress Scale (DASS-21) (37); 6) Strengths and Difficulties Questionnaire (SDQ) (38); 7) CRAFFT Screening Tool for Adolescent Substance Abuse (39); and 8) Acculturation, Habits, and Interests Multicultural Scale for Adolescents (AHIMSA) (40). Here we report results for the first seven questionnaires, as the data for the AHIMSA questionnaire has been published separately (41).

Psychometric properties for the measures used are well-established. The Child and Youth Resilience Measure-28 (CYRM-28) is a 28-item measure of child and youth resilience that measures individual, peer, family, and community resources implicated in resilience processes (42). Responses are scored using a 5-point scale ranging from *1 = does not describe me at all* to *5 = describes me a lot*, where higher scores indicate greater resilience. Factor analyses confirmed three latent variables (i.e., individual characteristics; relationships with caregivers; and contextual elements contributing to a sense of belonging). These inter-related variables have been shown to load onto a single resilience factor (42, 43). Internal reliability for the CYRM-28 is good, with Cronbach's α reported as ranging between .65 and .91 for the three latent variables (42).

The Connor-Davidson Resilience Scale (CD-RISC) is a widely used measure of youth trait resilience comprising 25 items measured on a 5-point scale ranging from *0 = not at all* to *4 = true nearly all of the time* (34). Original factor analysis revealed a five factor model where factor one referred to personal competence, tenacity, and high standards, factor two related to trusting one's instincts, tolerance of negative affect, and a strengthening effect of stress, factor three corresponded to acceptance of change and positive relationships, factor four to personal control, and factor five to spiritual influences (34). Internal reliability tests reported Cronbach's α for the full scale at 0.89 and item-total correlations ranged between 0.30 and 0.70. Test-retest reliability was good with an intraclass correlation coefficient of 0.87. Convergent validity was established through positive correlations between the CD-RISC and Kobasa's measure of hardiness (44) (Pearson *r* = 0.83, *P* < .0001) and the Sheehan Social Support Scale (SSS) (45) (Spearman *r* = 0.36, *P < .*0001). Negative correlations have been established with the Perceived Stress Scale (PSS-10) (46) (Pearson *r = −*0.76, *P < .*001), the Sheehan Stress Vulnerability Scale (SVS) (45) (Spearman *r = −*0.32, *P < .*0001), the Sheehan Disability Scale (SDS) (47) (Pearson *r = −*0.62, *P* < .0001) (34).

The Warwick-Edinburgh Mental Well-being Scale (WEMWBS) is a measure of wellbeing containing 14 positively worded items relating to positive attributes of mental health (e.g., item 1*: I've been feeling optimistic about the future*; item 5: *I've had energy to spare*), and is measured on a 5-point scale ranging from *1 = none of the time to 5 = all of the time.* The WEMWBS has been quantitatively validated in a student and adult UK population, as well as with Chinese and Pakistani ethnic minority groups in the UK (35, 48, 49). Initial assessment showed content validity was good with confirmatory factor analysis revealing a single *wellbeing* factor (GFI = 0.93, AGFI = 0.8, RMSEA = 0.055). Internal reliability tests of the scale reported Cronbach's α at 0.89; suggesting some item redundancy, item total correlations ranged from 0.52 and 0.80. Test-retest reliability for the WEMWBS was high (0.83) at 1 week and was found to discriminate between youth and adult populations well (48). The WEMWBS was also robust in measuring wellbeing in different ethnic populations (49).

The COMPAS Wellbeing Scale (COMPAS-W) is a composite measure of wellbeing comprising six subcomponents: composure during stress, own-worth, mastery over the environment, positivity, achievement and satisfaction with physical, psychological health and social relationships (36). The 26-item scale accounts for both hedonic (i.e., subjective) and eudaimonic (i.e., psychological) wellbeing constructs, with individual subscales measured using a 5-point scale ranging from *1 = strongly disagree* to *5 = strongly agree.* A composite wellbeing score is produced from the sum of the subscale scores. Construct validity for the COMPAS-W had been established through strong correlations with other measures of physical and psychological health behaviors, such as the World Health Organization Quality of Life scale (WHOQOL-BREF) (50), the Satisfaction with Life Scale (SWLS) (51), the Internal Control Index (ICI) (52), and the Emotion Regulation Questionnaire (53). Internal consistency for the COMPAS-W is strong (average *r* = 0.71; wellbeing *r* = 0.84) and test-retest reliability was robust across a 12-month period (average *r* = 0.62; wellbeing *r* = 0.82) (36).

The Depression Anxiety Stress Scale (DASS-21) is 21-item measure of state depression, anxiety, and stress (37). The DASS-21 is made up of three subscales for depression, anxiety, and stress respectively, which are each measured on a 4-point scale ranging from *0 = never to 4 = almost always*. DASS subscales have been shown to correlate well with other measures of depression and anxiety, such as the Beck Depression Inventory (BDI) (54) and the Beck Anxiety Inventory (BAI) (37, 55). The DASS has been found to differentiate clinical and non-clinical populations, as well as to discriminate between different clinical diagnostic groups (37, 56). Internal consistency for each subscale of the DASS-21 was good in a recent non-clinical sample (Cronbach's α was reported at .91, .80, and .84 for depression, anxiety, and stress, respectively) (57).

The Strengths and Difficulties Questionnaire (SDQ) is a screening tool used to assess the psychological adjustment of children and youth (38). The 25-item scale is made up of positively and negatively worded statements (e.g., item 1: *I am considerate of other people's feelings;* item 2: *I am restless, overactive and cannot stay still for long*). Participants respond to statements using a 3-point scale from *0 = not true*; *1 = somewhat true*; and *2 = certainly true*. Factor analysis supported a five-factor model, which included 1) emotional symptoms, 2) conduct, 3) hyperactivity-inattention, 4) peer relationships, and 5) pro-social behavior (38). Internal consistency was sound with Cronbach's α reported at 0.73 for the scale (38). In a U.S. sample, Cronbach coefﬁcients for subscale scores ranged from fair (α = 0.43) for peer problems to excellent for total difﬁculties (α = 0.83) and impairment scores (α = 0.80), and good to excellent for other subscales (α = 0.63–0.77) (58). Test-retest reliability was reasonable across a 4- to 6 month period (α = 0.62) (38).

The CRAFFT is a six-item screening test used to assess adolescents for substance use and abuse (39). Items ask directly about substance use (e.g., item 2: *do you ever use alcohol or drugs to relax, feel better about yourself or fit in?*) and require a simple *yes/no* response, with items summed for a final score. CRAFFT scores have been shown to correlate strongly with substance use classifications: 1) *no use*, 2) *occasional use*, 3) *problem use*, 4) *abuse,* and 5) *dependence* (Spearman's *r* = 0.72, *p* < .001), and scores above 2 are indicative of *problem use*, *abuse,* and *dependence* categories (59).

The Acculturation, Habits, and Interests Multicultural Scale for Adolescents (AHIMSA) is a measure of cultural identification in adolescents (40). AHIMSA comprises seven items and generates scores for four sub-scales: 1) country of residence orientation (assimilation), 2) other country orientation (separation), 3) both countries orientation (integration), and 4) neither country orientation (marginalization) (40). Three of the sub-scales correlated with subscales of a modified Acculturation Rating Scale for Mexican-Americans-II, with English language usage, providing initial evidence of construct validity (60). Internal consistency of the sub-scales was acceptable, with Cronbach's α ranging from 0.50 (marginalization) to 0.79 (assimilation and integration) (40).

### Procedure

The questionnaire was administered verbally (UK, New Zealand, South Africa) or completed by youth in hard copy (Canada, China) or *via* computer using Qualtrics survey software (Australia) (61); however, there were no differences in item content or ordering of items between the different administered versions. All research sites completed the full test battery, with the exception of the UK and South Africa for which participants did not complete the COMPAS-W Scale, and China for which participants did not complete the CRAFFT. In the UK, the WEMWBS wellbeing scale was preferred as a measure of wellbeing as this site had comparative data on this age group for another sample, and so the COMPAS-W was not administered to keep testing time minimal. Similarly in South Africa, the COMPAS-W was not administered due to ethical concerns that the administration of a second wellbeing questionnaire (in addition to the WEMWBS) would make the testing time too long. In China, the CRAFFT was not administered as it was not culturally acceptable to ask participants about the use of drugs and alcohol. Measures were translated and back-translated into Mandarin for the China cohort. All other country cohorts completed the questionnaire in English.

### Statistical Analysis

Data were collected from each research site and compiled into a single data file using the SPSS Statistics 24 package. Internal reliability of each questionnaire was evaluated across the sample and per site using Cronbach alpha.

Mean differences in trauma exposure frequency was evaluated between migrants *versus* non-migrants (controlling for site), as well as non-migrants *versus* internal and external migrants using univariate ANOVA. Variation in the type of event per group was examined using crosstabs chi-square analysis. This analysis was repeated to also compare differences between sites.

To then consider whether trauma exposure in the past year moderated the impact of mental health in migrants *versus* non-migrants, we examined the interaction effects of trauma exposure x migrancy status on mental health and resilience outcomes using univariate ANOVA, covarying for age, sex, site differences, and whole life trauma exposure. This analysis included a comparison of external *vs*. internal migrants *vs*. non-migrants. A *p* value significance threshold of 0.05 was adopted in all analyses.

## Results

### Internal Reliability

Internal reliability of each questionnaire across and within each site is shown in **Table 2**. Across the sample, all questionnaires showed high internal reliability. High internal reliability for most questionnaires was also evident within site, with some exceptions (e.g., lower estimates for the CYRM-28 and WEMWBS in the UK sample, likely due to its smaller sample size of 10; and lower estimates for the CRAFFT in the Australian, Canadian, and UK samples, likely due to increased variability in substance use/abuse within these sites).

**Table 2:** Internal reliability (Cronbach alpha) of each questionnaire by site.

Measure (no. of items)	Australia (*N* = 25)	Canada (*N* = 21)	China (*N* = 77)	New Zealand (*N* = 33)	South Africa (*N* = 28)	United Kingdom (*N* = 10)	Total (*N* = 194)
CYRM-28 (28)	0.831	0.869	0.926	0.929	0.874	0.333	**0.904**
CD-RISC (25)	0.811	0.896	0.932	0.925	0.916	0.792	**0.929**
WEMWBS (14)	0.829	0.877	0.922	0.896	0.840	0.537	**0.898**
COMPAS-W (26)	0.824	0.850	0.900	0.861	–	–	**0.883**
DASS-21 (21)	0.769	0.921	0.948	0.912	0.905	0.854	**0.931**
SDQ (20)	0.843	0.861	0.812	0.862	0.811	0.846	**0.823**
CRAFFT (6)	0.480	0.310	–	0.782	0.727	0.107	**0.721**

CYRM-28, Child and Youth Resilience Measure; CD-RISC, Connor-Davidson Resilience Scale; WEMWBS, Warwick-Edinburgh Mental Well-being Scale; COMPAS-W, COMPAS-W Wellbeing Scale; DASS-21, Depression, Anxiety, Stress Scale; SDQ, Strengths and Difficulties Questionnaire; and CRAFFT, CRAFFT Screening Tool for Adolescent Substance Abuse. **“–” reflects missing data due to China not administering the CRAFFT, and South Africa/United Kingdom not administering the COMPAS-W.

### Mean Differences in Trauma Exposure

**Figure 1** presents the frequency (percentage) of types of childhood traumatic events reported across the sample, for both the past year and lifetime. Mean total events reported for the past year and lifetime were 1.26 (± 1.53) and 2.54 (± 1.85), respectively.

We next considered differences in traumatic events reported in migrant *versus* non-migrant groups, controlling for site differences. There were no significant differences between migrants and non-migrants in the total mean traumatic events reported over the *lifetime* (F = 3.70, p = .056). There was however a significant difference in the total mean traumatic events reported in the *past year* (F = 5.55, p = .019), with migrants reporting a higher mean number of events (M = 1.43, SD = 1.62) than non-migrants (M = 0.71, SD = 0.97). There were also differences between types of trauma reported by migrants and non-migrants. Relative to non-migrants, migrants reported more episodes of combat experience in war (NM: 0%, M: 13% exposure, p = .010) and death of a family member or close friend (NM: 44%, M: 62% exposure, p = .034) in their lifetime, plus more episodes of death of a family member or close friend than non-migrants in the past year (NM: 16%, M: 34%, p = .048).

**Figure 2:**
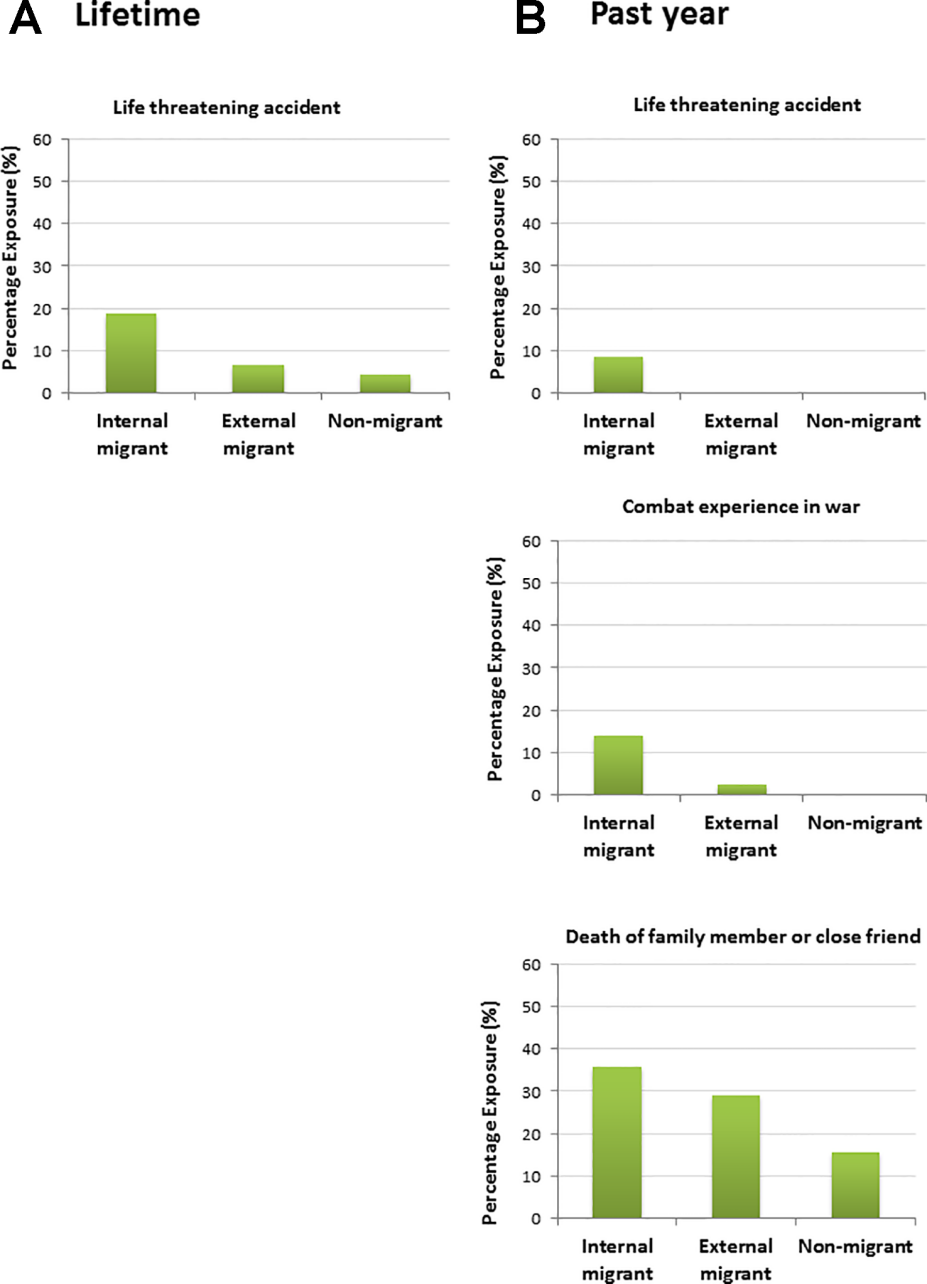
Percentage exposure (% of “yes” responses) for significant differences by migrancy groups for total traumatic events reported during **(A)** the lifetime and **(B)** the past year.

We then considered whether the differences in traumatic events reported in migrants *versus* non-migrants varied when stratifying by internal *versus* external migrants. There were no significant differences between migrants (internal *vs*. external) and non-migrants in the total mean traumatic events reported over the *lifetime* (F = 2.24, p = .110). There was however a significant difference in the total mean traumatic events reported in the *past year* (F = 4.66, p = .011), with internal migrants reporting a higher mean number of events (M = 1.59, SD = 1.74) than external migrants (M = 1.05, SD = 1.26) and non-migrants (M = 0.71, SD = 0.97). There were also differences between exposure for certain types of events. For lifetime events (see **Figure 2A**), internal migrants reported a higher number of life threatening accidents (19%) relative to external migrants (7%) and non-migrants (4%, p = .009). For past year events (**Figure 2B**), internal migrants reported a higher number of combat/war experiences relative to external migrants and non-migrants (M^I^: 14%, M^E^: 3%, NM: 0%, p = .015), a higher number of life threatening accidents (M^I^: 9%, M^E^: 0%, NM: 0%, p = .030), and death of a close family member or friend (M^I^: 36%, M^E^: 29%, NM: 16%, p = .039).

**Figure 3:**
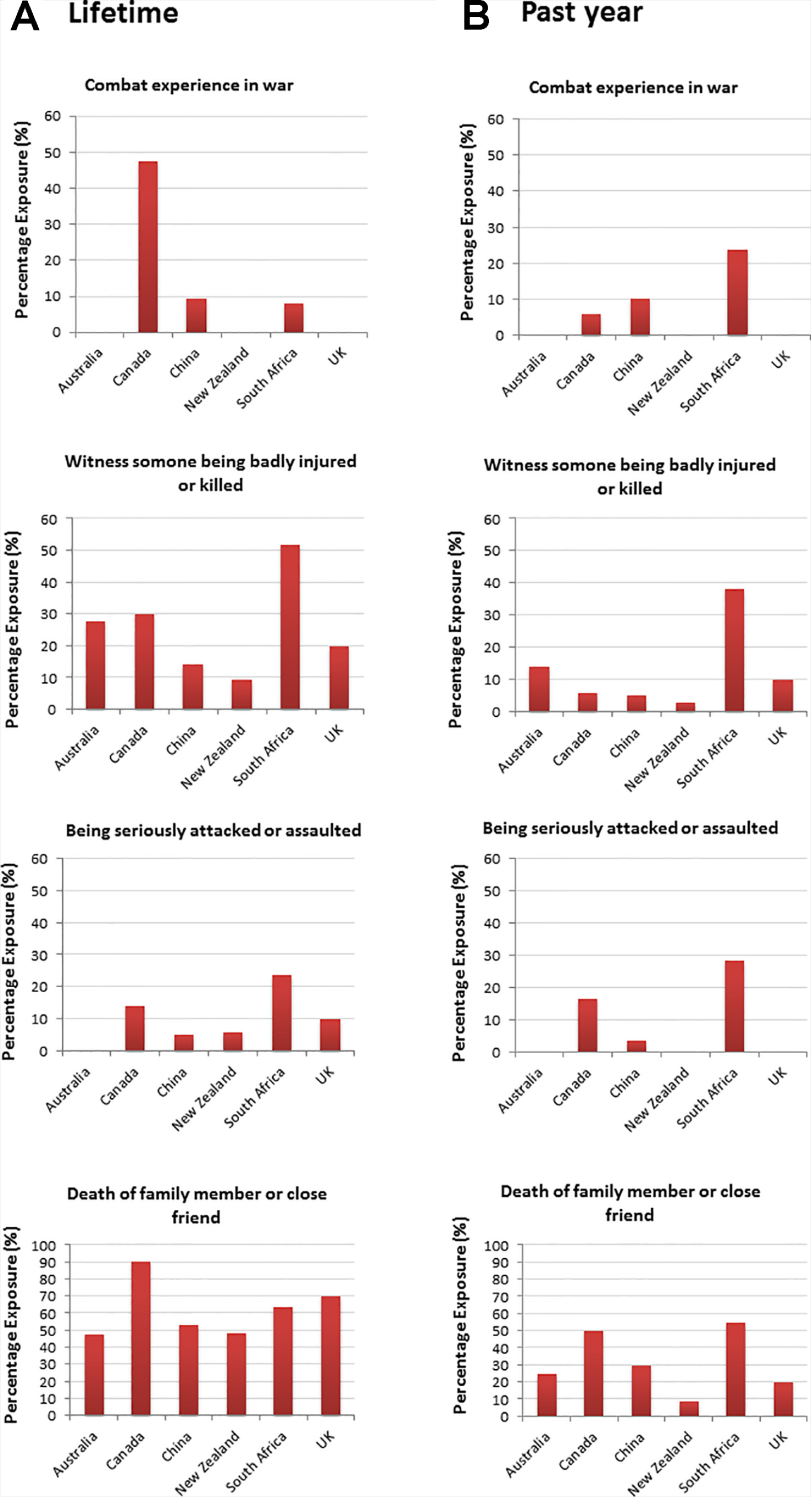
Percentage exposure (% of “yes” responses) for significant site differences by total traumatic events reported during **(A)** the lifetime and **(B)** the past year. For **(B)**, site differences were also found for “life-threatening accidents” (China: 5%, South Africa: 20% percentage exposure), and “threatened by a weapon/held captive/kidnapped” (Australia: 100%, China: 3.4%, South Africa: 15% percentage exposure) (not presented here).

We then examined reported traumatic event differences between the sites. There were no significant differences between sites in the total mean traumatic events reported over the *lifetime* (F = 1.95, p = .088). There was a significant difference in the total mean traumatic events reported in the *past year* (F = 5.25, p < .0001), with South African youth reporting a higher mean number of events (M = 2.43, SD = 2.13) relative to every other site: Australia (M = 0.80, SD = 1.08, p < .0001), Canada (M = 1.24, SD = 1.58, p = .005), China (M = 1.29, SD = 1.47, p < .0001), New Zealand (M = 0.73, SD = 0.84, p < .0001), and the UK (M = 0.80, SD = 0.92, p = .003). There were also differences between sites for exposure to specific types of traumatic events reported during the *lifetime* and *past year.* For lifetime events (see **Figure 3A**), significant differences between sites were evident for combat/war exposure (p = .0001), witnessing serious injury/murder (p = .001), attack/assault (p = .029), and death of family member/close friend (p = .023). There were also significant site differences for past year events (see **Figure 3B**) for combat/war exposure (p = .032), life threatening accident (p = .023), witnessing injury/murder (p = .001), attack/assault (p = .001), being threatened by a weapon, held captive or kidnapped (p = .0001), and death of family member or close friend (p = .005).

### Main and Interacting Effects of Trauma and Migrancy on Wellbeing and Mental Health Outcomes

To then consider whether trauma exposure in the past year moderated the impact of mental health in migrants *versus* non-migrants, we examined the interaction effects of trauma exposure x migrancy status on mental health and resilience resources using univariate ANOVA, covarying for any age, sex, site differences, and whole life trauma exposure effects. We also considered the added comparison of external migrants *vs*. internal migrants *vs*. non-migrants to evaluate whether type of migrancy had a differential impact.

**Figure 4:**
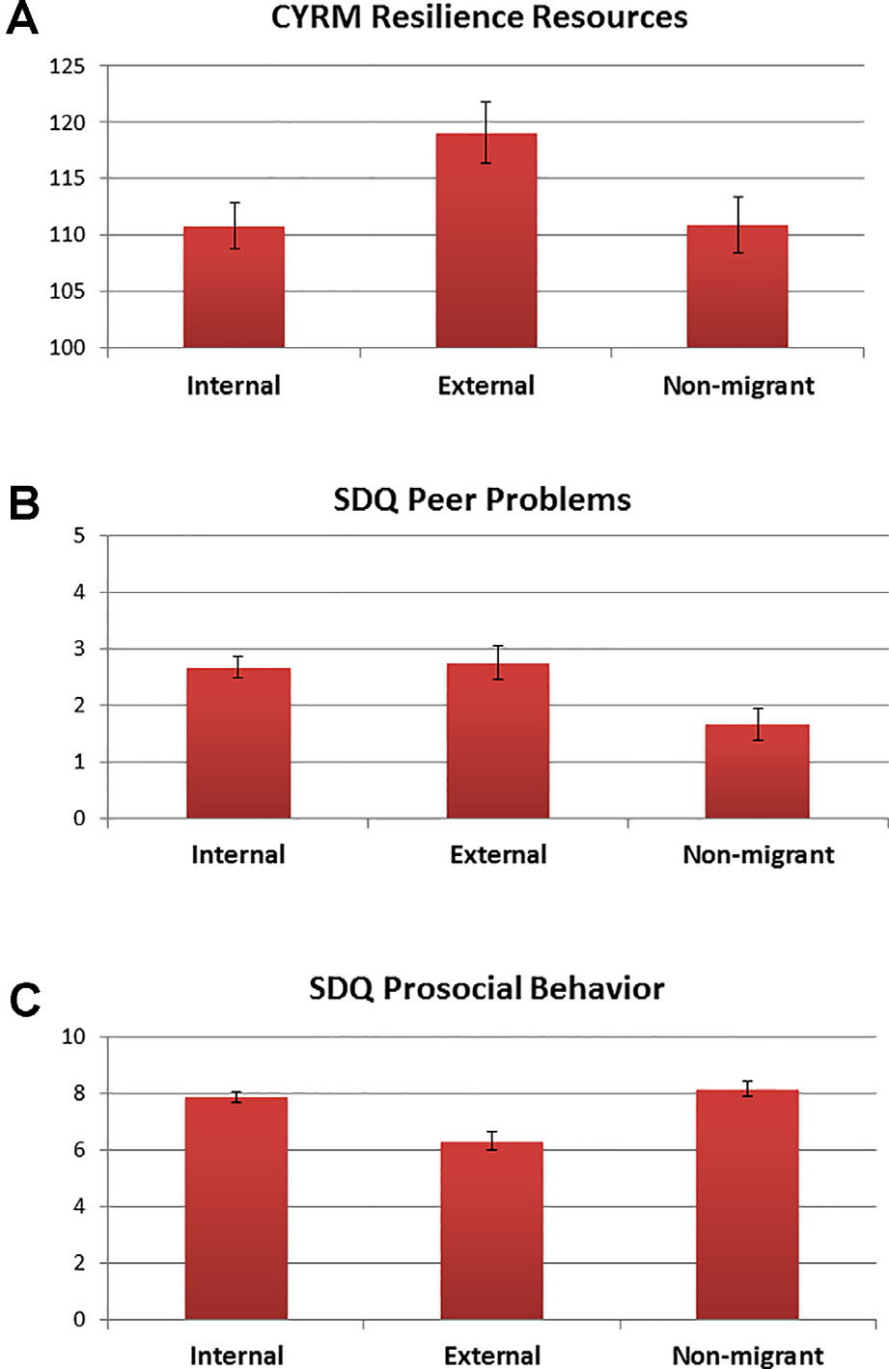
Means and SE bars for significant main effects of migrancy for **(A)** Child and Youth Resilience Measure (CYRM) resilience resources, **(B)** Strengths and Difficulties Questionnaire (SDQ) peer problems, and **(C)** SDQ prosocial behavior.

There was no significant difference between migrants and non-migrants in their resilience resources as measured by the CYRM-28. When considering types of migration, a main effect was found for migrancy (F = 3.37, df = 2, p = .037), whereby external migrants had a significantly higher CYRM-28 resilience score (M = 119.03, SE = 2.73) compared to internal migrants (M = 110.83, SE = 2.01; see **Figure 4A**). There was no main effect of trauma, or trauma by migrancy effects, on the CYRM-28.

**Figure 5:**
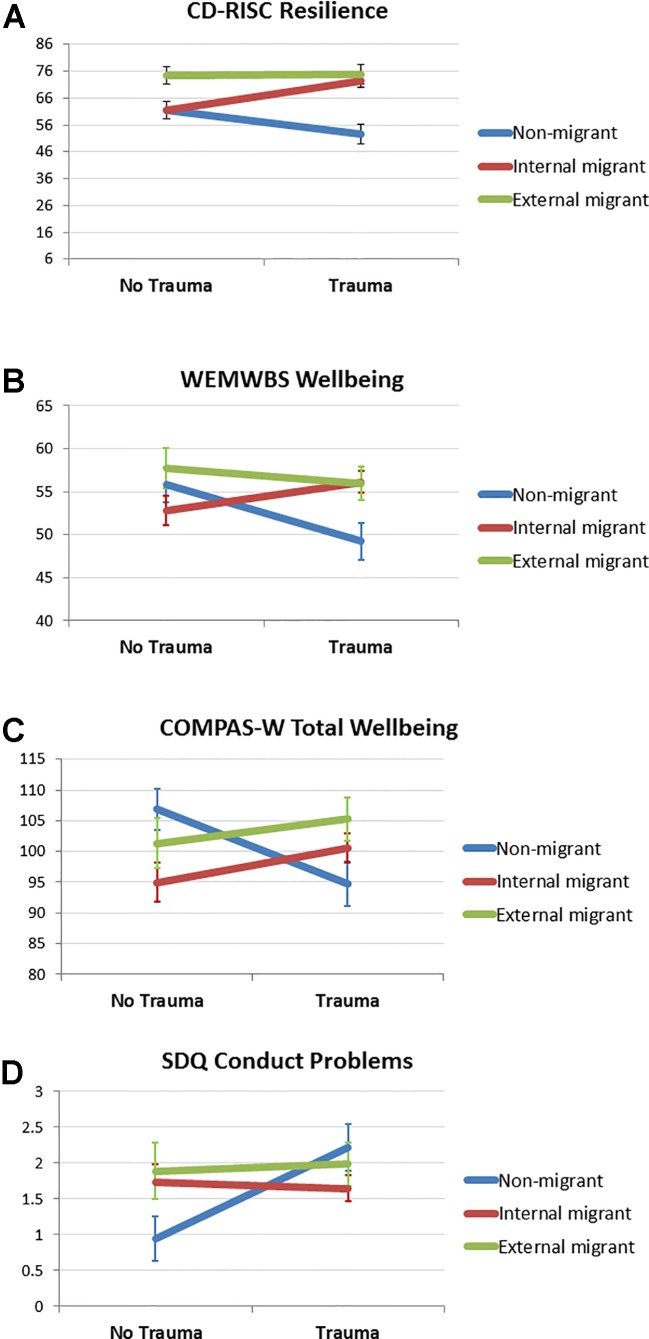
Means and SE bars for significant interaction effects of trauma by migrancy for **(A)** Connor-Davidson Resilience Scale (CD-RISC) resilience scores, **(B)** Warwick-Edinburgh Mental Well-being Scale (WEMWBS) wellbeing scores, **(C)** COMPAS Wellbeing Scale (COMPAS-W) wellbeing scores, and **(D)** Strengths and Difficulties Questionnaire (SDQ) conduct problems.

For the CD-RISC resilience measure, there was a significant main effect for migrancy (F = 21.37, df = 1, p < .0001), whereby migrants demonstrated higher resilience (M = 69.92, SE = 1.52) than non-migrants (M = 56.33, SE = 2.44). When considering types of migration, a main effect was again found (F = 13.15, df = 2, p < .0001), whereby external migrants had a significantly higher resilience score (M = 74.64, SE = 2.68) compared to internal migrants (M = 66.86, SE = 1.99). There was no main effect of trauma on CD-RISC scores, yet there was a trauma by migrancy effect (F = 8.31, df = 1, p = .005). Higher resilience scores were evident in migrants exposed to trauma than non-trauma, whereas lower resilience scores were evident in non-migrants exposed to trauma *vs*. non-trauma. Moreover, trauma-exposed migrants showed higher resilience scores than trauma-exposed non-migrants. When considering types of migration, a trauma by migrancy effect was also evident (F = 5.61, df = 2, p = .005). External migrants showed higher resilience than internal migrants in the non-trauma group, but there were no differences between external and internal migrants in the trauma-exposed group (**Figure 5A**).

No significant main effects of migrancy or trauma were evident for wellbeing when measured using the WEMWBS. There were also no effects of migrancy when considering different types of migration. A significant interaction effect of trauma by migrancy was however evident (F = 6.43, df = 1, p = .012). Migrants and non-migrants showed similar wellbeing scores in the absence of trauma, yet in the trauma-exposed group, non-migrants (M = 48.93, SE = 2.16) showed significantly lower wellbeing than trauma-exposed migrants (M = 56.19, SE = 1.06). This interaction effect was also significant when considering types of migrants (F = 4.29, df = 2, p = .015). Again, no group differences were evident in wellbeing in the absence of trauma, yet in the trauma-exposed group, it was the non-migrants (M = 49.23, SE = 2.17) which showed lower wellbeing than the internal migrants (M = 56.13, SE = 1.27) or external migrants (M = 55.92, SE = 1.97; **Figure 5B**).

Similar to the results above, no significant main effects of migrancy or trauma were evident for total wellbeing when measured using the COMPAS-W scale. A significant interaction effect of trauma by migrancy was however evident (F = 10.825, df = 1, p = .001). In the absence of trauma exposure, non-migrants (M = 106.98, SE = 3.29) showed higher levels of wellbeing than migrants (M = 97.41, SE = 2.45); yet in the trauma-exposed group, non-migrants (M = 94.10, SE = 3.52) showed reduced levels of wellbeing than trauma-exposed migrants (M = 102.08, SE = 1.98). This interaction effect was also significant when considering types of migrants (F = 5.22, df = 2, p = .007). In the absence of trauma exposure, non-migrants (M = 106.83, SE = 3.28) showed higher levels of wellbeing than internal migrants in particular (M = 94.96, SE = 3.20) with external migrants showing no differences between the other two groups (M = 101.30, SE = 4.06). Yet, when trauma-exposed, the wellbeing scores of the two migrant groups appeared unaffected (IM: M = 100.55, SE = 2.37; EM: M = 105.26, SE = 3.52), whereas the non-migrants showed a reduction in wellbeing when trauma-exposed (M = 94.72, SE = 3.54; **Figure 5C**). A similar pattern of significant trauma x migrancy interaction effects were also found for the COMPAS-W subscales Composure, Mastery, Positivity, Achievement, and Satisfaction (see **Supplementary Materials**).

In respect to depression, anxiety, and stress as measured by the DASS-21, there were no significant main or interaction effects of trauma or migrancy in terms of total general distress or depression, anxiety, and stress subscores. There were also no significant main or interaction effects of trauma or migrancy for self-reported substance-related risks and problems as measured by CRAFFT.

When considering behavioral problems measured by the SDQ, several main and interaction effects were evident. First, we identified two main effects of migrancy for peer problems (F = 10.30, df = 1, p = .002) and prosocial behavior (F = 7.44, df = 1, p = .007), for which migrants showed higher peer problems (M = 2.70, SE = 0.15) and lower prosocial behavior (M = 7.4, SE = 0.16) than non-migrants (peer problems: M = 1.66, SE = 0.28; prosocial: M = 8.34, SE = 0.29). When considering types of migrancy, these main effects were again significant for peer problems (F = 5.16, df = 2, p = .007) and prosocial behavior (F = 12.40, df = 2, p < .0001). In this case, both internal (M = 2.67, SE = 0.19) and external migrants (M = 2.76, SE = 0.31) showed higher peer problems than non-migrants (M = 1.67, SE = 0.28; **Figure 4B**). In addition, external migrants showed the lowest prosocial behavior (M = 6.33, SE = 0.30), followed by internal migrants (M = 7.89, SE = 0.20), with non-migrants showing the highest level of prosocial behavior (M = 8.16, SE = 0.28; **Figure 4C**). Second, we identified a main effect of trauma for conduct problems (F = 6.98, df = 1, p = .022), whereby trauma exposed participants showed higher conduct problems (M = 1.96, SE = 0.18) than non-trauma exposed participants (M = 1.35, SE = 0.19). There was also a trauma by migrancy effect for conduct problems (F = 6.98, df = 1, p = .009), whereby in the absence of trauma exposure, non-migrants showed fewer conduct problems (M = 0.92, SE = 0.30) than migrants (M = 1.78, SE = 0.20). Yet, in the presence of trauma exposure, migrants showed no difference in conduct problems (M = 1.74, SE = 0.16), whereas non-migrants showed an increase in conduct problems (M = 2.18, SE = 0.33). This interaction effect for conduct problems was also significant when considering types of migrancy (F = 3.59, df = 2, p = .030), whereby non-migrants showed fewer conduct problems in the absence of trauma exposure (M = 0.94, SE = 0.31) than both internal migrants (M = 1.73, SE = 0.24) and external migrants (M = 1.89, SE = 0.40), but in the presence of trauma exposure, non-migrants showed similar levels of conduct problems (M = 2.22, SE = 0.33) to internal migrants (M = 1.65, SE = 0.19) and external migrants (M = 1.99, SE = 0.31; **Figure 5D**).

## Discussion

This aim of this study was to use our pilot data to explore the impact of site, migrancy, and trauma exposure on resilience, wellbeing, and mental health among migrant and non-migrant adolescents aged 10–17 in multiple countries where there are high rates of internal and external migration. Our key research questions aimed to clarify 1) whether the measures of resilience, wellbeing, mental health, and behavior were reliable across country sites, 2) whether differences were apparent between migrant and non-migrant adolescents and between sites in trauma exposure, 3) whether there were differences between migrant and non-migrants in behavioral and mental health outcomes, and 4) how trauma and migration was related to resilience, behavior and wellbeing.

First, we have shown that the structured questionnaire administered in the current study was feasible and acceptable in this age group, and had good validity when used in different settings with youth of the same age. All questionnaires showed high internal reliability across the total sample, with some small variability in estimates for specific sites likely due to smaller sample sizes and variability in health behaviors for specific subsamples (particularly for the UK sample with N = 10).

With regard to the second question, a number of key differences in trauma exposure were found for migrants and non-migrants, and by site. Generally speaking, migrants reported a higher mean number of traumatic events in the past year than non-migrants, with internal migrants reporting the most events. The types of events that varied the most between migrant groups were exposure to life-threatening accidents, combat/war experience, and death of a family member or close friend. When we considered variation by site, South African youth reported a higher mean number of events relative to all other country sites. Importantly, the effects of migrancy were significant despite including site as a covariate, so the effects were not specific to any country of origin in particular but rather by virtue of migrancy status specifically.

Thirdly, we identified a number of differences between the migrant groups in terms of mental health and behavioral outcomes. Migrant youth reported higher CD-RISC resilience scores than non-migrants, yet they also reported more behavioral problems in terms of higher SDQ peer problems and lower prosocial behaviors. However, when we considered type of migrancy, the external migrants showed the higher resilience scores yet lower SDQ prosocial behavior scores than the internal migrants and non-migrants. External and internal migrant groups showed no difference in the SDQ peer problems (both higher than non-migrants). Together, this suggests that perhaps the external migrants showed higher resilience than internal migrants because they were able to move away from the trauma (by moving countries), whereas internal migrants may not have been able to move “away” from the adversity. This argument is strengthened by the fact that the internal migrants showed the highest percentage of past year traumatic events due to combat/war, life threatening accidents, and death of a family member/friend in particular, suggesting the adversity may still be present or having an impact. In contrast to these findings for resilience, migrants did however report more behavioral problems and less prosocial behaviors toward peers. This effect is likely a reflection of challenges that youth would experience when entering and assimilating into a new school system; in particular, the larger challenge of creating new peer networks within a new cultural environment, and often in another primary language for many external migrants.

Finally, we found that the presence of trauma modulated the mental health and behavioral outcomes of non-migrants in particular, rather than migrants who showed no differences in scores when comparing trauma and non-trauma exposed groups. For instance, in terms of CD-RISC resilience scores, migrants had higher resilience than non-migrants in the presence of trauma. This effect was apparent in both internal and external migrant groups, although in the absence of trauma, external migrants still showed higher resilience scores. Together, this suggests that migrant youth, particularly external migrants, show a resilient response to adversity, especially in the presence of trauma or hardship. As this is cross-sectional data, it is difficult to delineate whether this effect is due to these migrant groups being able to move “away” from the trauma and hence they then feel they have more resilience resources, or because they had an inherent disposition of stronger adaptation or sense of agency which underscored the motivation for them (and their family) to change their living environment and move away. For wellbeing (measured using the WEMWBS and COMPAS-W scales), the migrant youth (both internal and external) showed higher levels of wellbeing than non-migrants in the presence of trauma. This effect may again reflect the increased positive mental health state of migrant youth compared to non-migrant youth given they were able to move away from the most recent trauma. Finally, in terms of SDQ conduct problems, the presence or absence of trauma did not appear to impact migrant conduct behavior for both internal and external migrants. Yet non-migrants showed lower conduct problems in the absence of trauma, but an increase in conduct problems in the presence of trauma. Overall, these effects suggest that the mental health behaviors of migrants appeared to be unaffected by the presence or absence of trauma, whereas non-migrants show significant detriments in resilience, wellbeing, and conduct problems in the presence of trauma. Migrant youth do however appear to demonstrate more peer problems than non-migrant youth and less prosocial behaviors for external migrants in particular.

Previous studies focusing on the mental health of migrant youth have either focused on refugee youth in particular, with limited direct comparisons of mental health outcomes to immigrant and non-migrant comparative groups, and/or broadly defined immigrant groups with limited consideration of time since migrancy, generational effects and/or cross-cultural differences (25, 27–30). Nonetheless, these studies have identified a number of protective factors for mental health including psychological wellbeing of the parents/guardians, peer and social support, religious beliefs, and integration into the host community, whereas risk factors of poorer mental health outcomes included trauma exposure, parental exposure to violence, loss of parent(s), limited family support, violence and discrimination in host country, and feeling disconnected to school and neighborhood (25, 27, 29). In contrast to some of these effects, our findings suggest that trauma-exposed migrant youth are more resilient and demonstrate higher levels of wellbeing in comparison to their non-migrant trauma-exposed peers. The presence of trauma had no impact on the conduct behaviors of migrant youth relative to non-migrants who *were* more significantly impacted by trauma exposure. Migrant youth did however demonstrate more peer problems and less prosocial behaviors than their non-migrant trauma-exposed peers, which is consistent with previous reports of increased behavioral problems in refugee youth (27). Given the current sample included both immigrants and refugee migrant youth, it is possible that the role of trauma in the current study showed a differential impact to previous studies focusing on refugee youth alone. Indeed, in the recent study comparing mental health outcomes of refugee *versus* immigrant youth aged 11–13 years in Canada, it was the refugee youth that demonstrated significantly higher emotional problems, aggressive behavior, and pre-post-migration trauma than immigrant youth (27). However, as participants needed to be living in Canada for 10 years or less, it is unclear whether any differences varied with the recency of migration. It is therefore worthwhile to compare these migrant subgroups over time. Examining these associations longitudinally will help determine whether these higher levels of resilience and wellbeing in migrant youth are sustained over time, or whether they are a short-term outcome from possibly moving away from the trauma. Recent studies in fact suggest that factors such as postarrival discrimination or acculturative stress can cause additional harm on mental health outcomes, whereas feeling welcomed at school can mitigate against mental and behavioral problems (27, 29). Thus, it would be important to confirm whether the behavioral problems linked to peers and prosocial behaviors is alleviated with time as the young people become more acquainted with their new school environment and peer networks, or whether this worsens and has a subsequent detrimental impact on their psychological and cognitive development.

The current study was an international pilot study conducted across a range of contexts in high and middle income countries, including both external and internal migrant adolescents and non-migrant adolescents. The migrants included refugees and economic migrants. To our knowledge this is the first reported study of its kind. The study also included wellbeing and resilience findings in addition to risk/vulnerability outcomes. As the study was cross-sectional and limited by sample size in each country, this restricted some statistical analyses and comparisons that could be made (e.g., refugee *vs*. economic migrant adolescents). The limited sample sizes of some specific sites may have also impacted the reliability of some measures, as reported earlier. Thus, it would be worthwhile to replicate these outcomes in a larger sample, controlling for multiple comparisons to minimize potential false positive reporting. Some questions were also not culturally acceptable in some sites, including for instance those asking about the use of drugs and alcohol in China, so had to be omitted. This limited the inclusion of some sites in the analyses, but is an issue that needs to be acknowledged in future international trials. Another limitation of this study is that recruitment was based on voluntary participation, so self-selecting participants (particularly some migrant adolescents) may have been more resilient to begin with. It would therefore be important to confirm the current findings in a larger and even more diverse sample of adolescents.

In conclusion, we found that, with some adjustment for cultural sensitivity, the current questionnaire included a reliable set of measures to use in an international study of migrant and non-migrant adolescent populations. Some interesting group differences in mental health outcomes were observed between migrants and non-migrants in the presence/absence of trauma exposure, which may open up avenues for future research. Our findings indicate that promoting mental health and wellbeing is an important strategy to implement for all young people, particularly those recovering from adversity, migrant or not. There is a need for further research with larger prospective sample sizes to investigate levels of resilience and mental health behaviors in migrant adolescents over time, and ways of promoting increased peer support networks in schools, as well as resilience in trauma-exposed young people, regardless of migrancy status.

## Ethics Statement

Ethics approval was sought and gained from the respective sites according to the local Human Research Ethics Committee processes (Australia; University of New South Wales Human Research Ethics Committee: HC15672; Canada; Dalhousie University Social Sciences and Humanities Research Ethics Board: REB 2015-3666; China; Chinese University of Hong Kong; New Zealand; The University of Auckland Human Ethics Committee: 015968; South Africa: North-West University Humanities and Health Research Ethics Committee: NWU-HS-2015-0234; United Kingdom; University of Bristol Faculty of Medicine Research Ethics Committee: ref 2016/26061). Written and/or verbal information was provided to all participants. Informed verbal and/or written consent was obtained from parents and informed verbal or written assent was gained from youth.

## Author Contributions

JG coordinated the study across the six country sites, including the Australian site, analyzed and interpreted the data, and wrote parts of the paper and revisions. RA collected data for the Australian site, completed data entry, and wrote parts of the paper. AE coordinated the data collection in the UK, and assisted with data interpretation and drafting of the paper. KF assisted with the coordination of the Australian study, data interpretation, and drafting of the paper. KH and MU coordinated the data collection in Canada, assisted with data interpretation and paper editing. AM-J assisted with data collection coordination in the UK, data interpretation, and paper editing. SR was the academic lead for the WUN group, and assisted with data interpretation and paper editing. LT coordinated the study in South Africa and assisted with data interpretation and paper editing. TW coordinated the study in New Zealand, wrote parts of the paper, and assisted with data interpretation and paper editing. QW coordinated the study in Hong Kong and assisted with data interpretation and editing.

## Funding

JG was supported by a National Health & Medical Research Council (NHMRC) Career Development Fellowship (APP1062495). RA was supported by an APA PhD scholarship. AE was funded by the University of Bristol. AM-J was funded by the University of York World wide Universities Network (WUN) funding for travel to project meetings in South Africa and Maastricht and Department of Health Sciences for time devoted to the project. SR was funded by the University of Cape Town. Meeting travel and pilot work was supported by LT's National Research Foundation Incentive Funding (IFR2011041100058). MU was funded by the Social Sciences and Humanities Research Council of Canada (885-2008-1000). TW was funded by the University's World wide Universities Network (WUN) funding and the University of Auckland's postgraduate funding. 

## Conflict of Interest

JG was a stockholder in MAP Corp. Pte Ltd.

The remaining authors declare that the research was conducted in the absence of any commercial or financial relationships that could be construed as a potential conflict of interest.
