# Supplementary material for: Trauma, Resilience, and Mental Health in Migrant and Non-Migrant Youth: An International Cross-Sectional Study Across Six Countries
Source: Front Psychiatry. 2020 Mar 9;10:997. doi: 10.3389/fpsyt.2019.00997 (PMC7073329; doi:10.3389/fpsyt.2019.00997)
Supplement: Supplementary file 1 [file Image_1.pdf]

Supplementary Figure 1:

Means and SE bars for significant interaction effects of trauma by migrancy for COMPAS-W subscales: (a) Composure, (b) Mastery, (c) Positivity, (d) Achievement, and (e) Satisfaction.

a

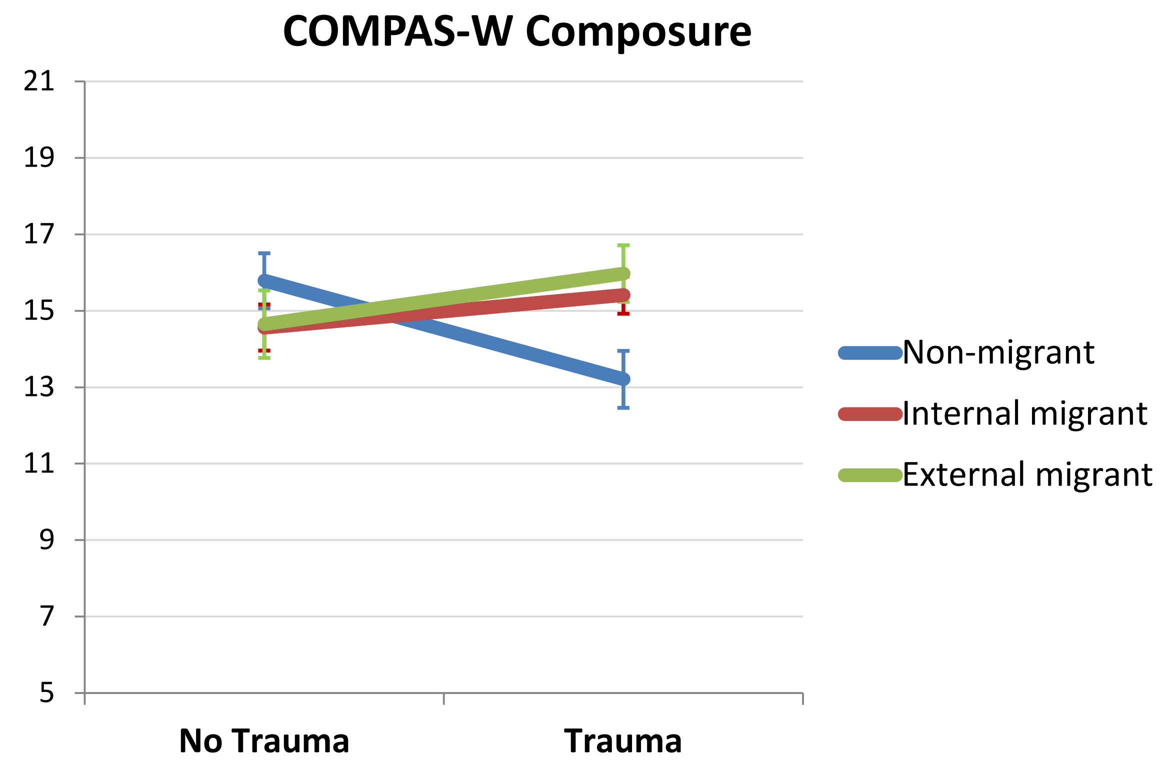

b

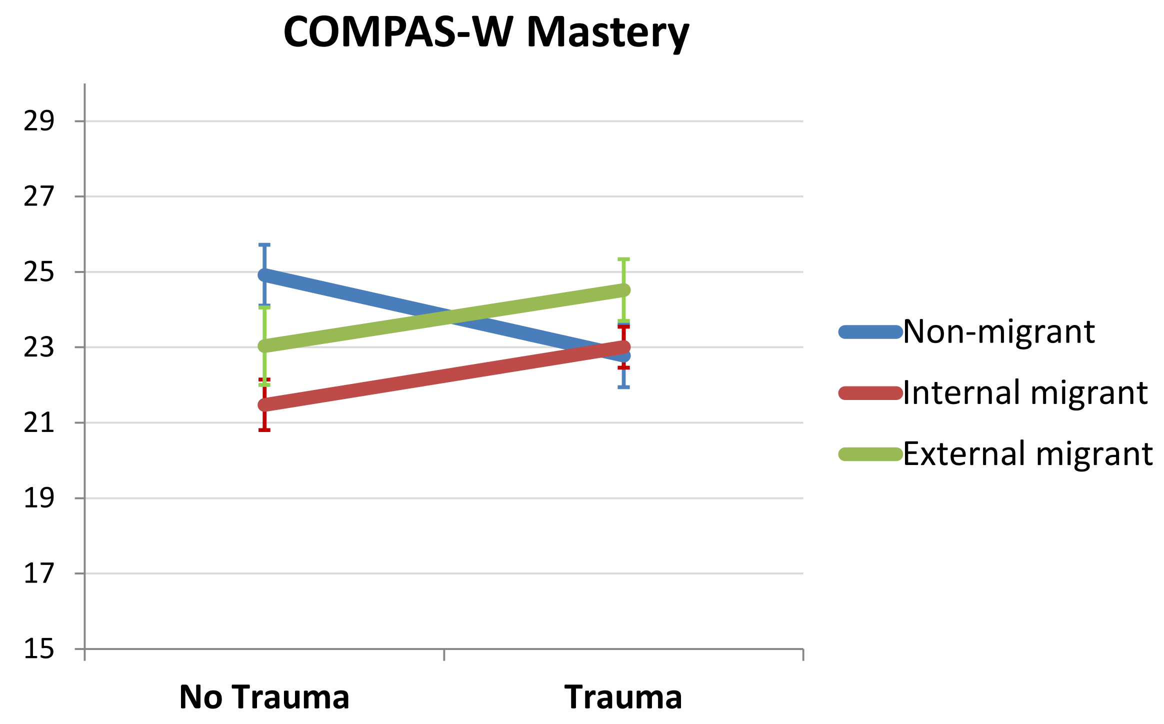

c

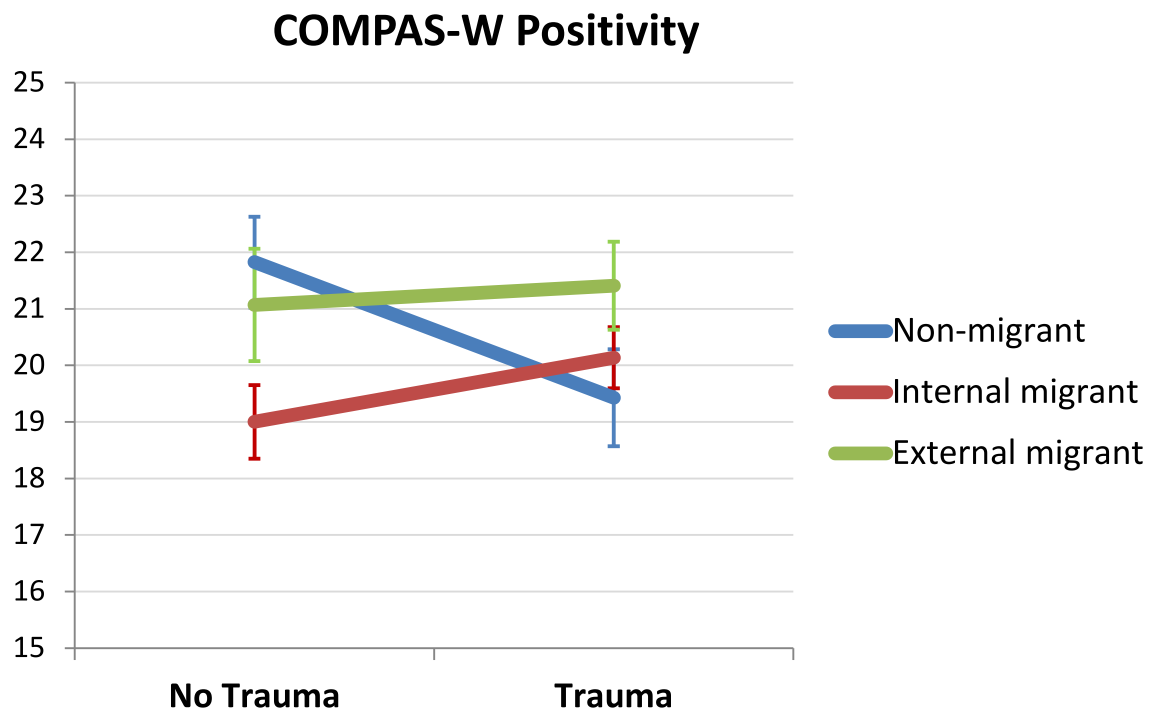
COMPAS-W Positivity
25
24
23
22
21
20
19
18
17
16
15
No Trauma
Trauma
Non-migrant
Internal migrant
External migrant

d

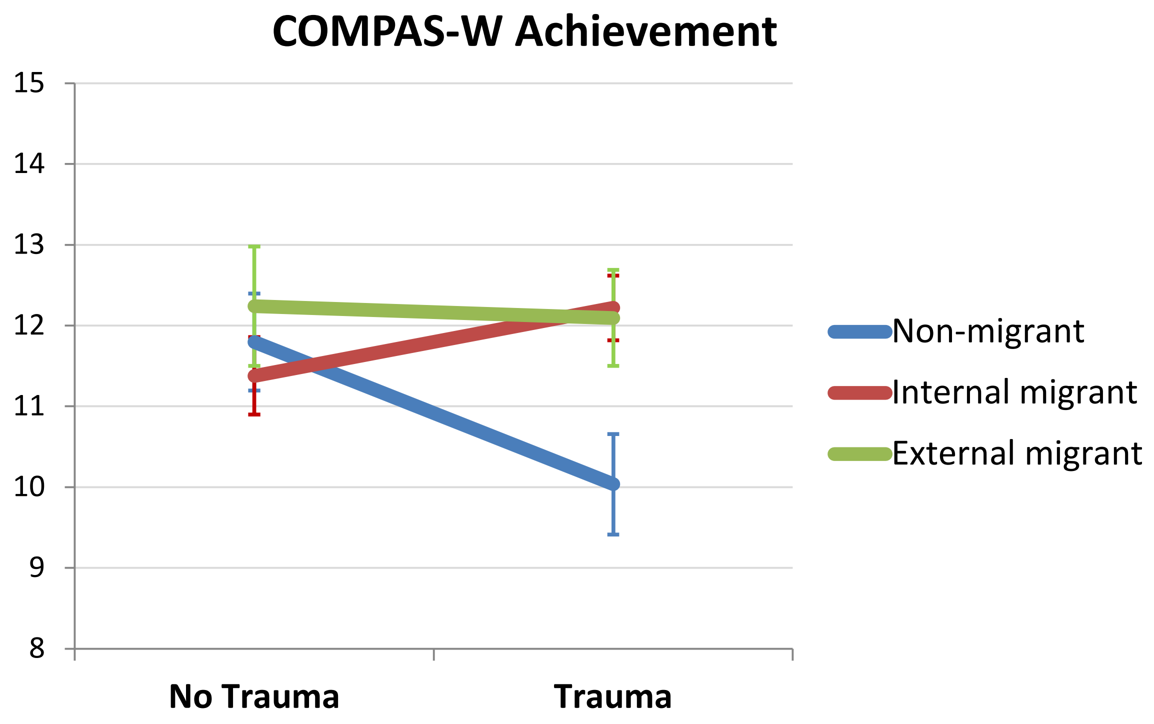
COMPAS-W Achievement
15
14
13
12
11
10
9
8
No Trauma
Trauma
Non-migrant
Internal migrant
External migrant

e

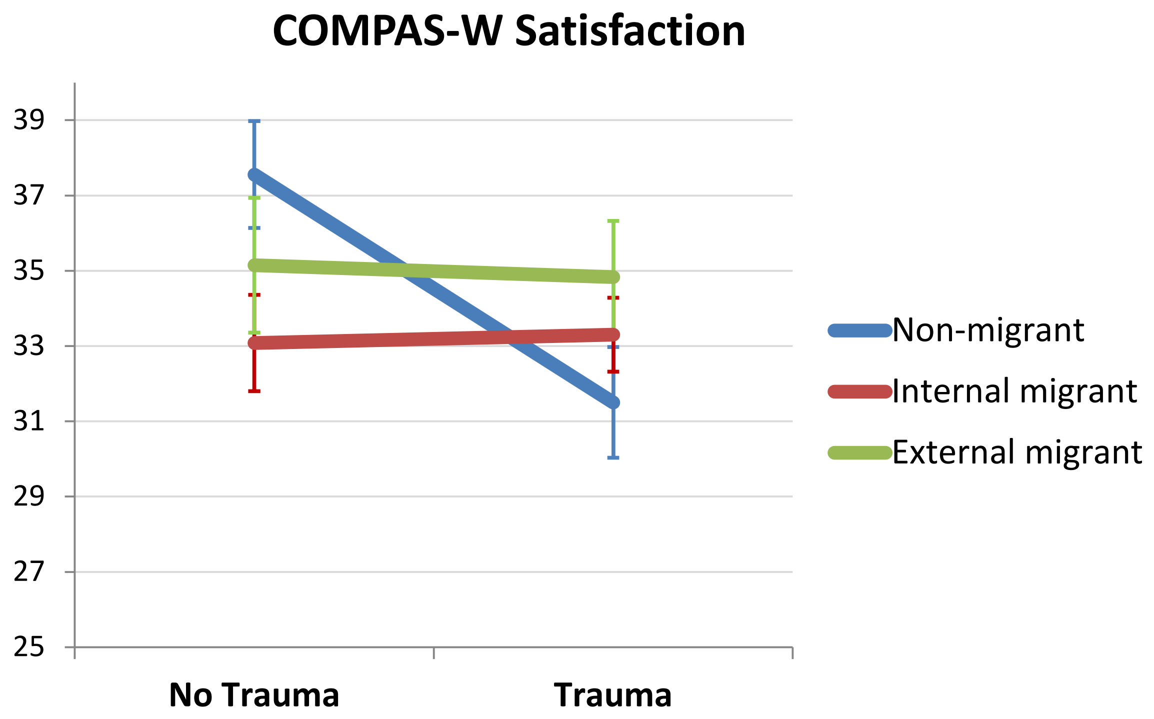

COMPAS-W Satisfaction
39
37
35
33
31
29
27
25
No Trauma
Trauma
Non-migrant
Internal migrant
External migrant
