# Supplementary material for: Trauma, Resilience, and Mental Health in Migrant and Non-Migrant Youth: An International Cross-Sectional Study Across Six Countries
Source: Front Psychiatry. 2020 Mar 9;10:997. doi: 10.3389/fpsyt.2019.00997 (PMC7073329; doi:10.3389/fpsyt.2019.00997)
Supplement: Supplementary file 2 [file Table_1.docx]

**Supplementary Table 1.**

Main and interaction effects of migrancy x trauma for the COMPAS-W subscales

| **COMPAS-W Measure** | **Main effect:**  **Migrancy** | | **Main effect:**  **Trauma Exposure** | | **Interaction Effect:**  **Migrancy x Trauma** | |
| --- | --- | --- | --- | --- | --- | --- |
|  | **F** | **p value** | **F** | **p value** | **F** | **p value** |
| Composure | 0.54 | 0.582 | 0.05 | 0.819 | 5.20 | **0.007** |
| Own-worth | 0.07 | 0.929 | 0.27 | 0.602 | 2.98 | 0.055 |
| Mastery | 2.50 | 0.086 | 0.20 | 0.658 | 4.13 | **0.018** |
| Positivity | 2.14 | 0.122 | 0.22 | 0.639 | 3.51 | **0.033** |
| Achievement | 1.98 | 0.143 | 0.54 | 0.466 | 3.51 | **0.033** |
| Satisfaction | 0.76 | 0.471 | 2.94 | 0.089 | 3.80 | **0.025** |

*Note.* df = 2, 1, 2 for main and interaction effects, respectively. Bolding indicates significant effects.
